# Prevalence, and Intellectual Outcome of Unilateral Focal Cortical Brain Damage as a Function of Age, Sex and Aetiology

**DOI:** 10.1155/2002/634764

**Published:** 2002-04-12

**Authors:** C. M. J. Braun, I. Montour-Proulx, S. Daigneault, I. Rouleau, S. Kuehn, M. Piskopos, G. Desmarais, F. Lussier, C. Rainville

**Affiliations:** ^1^ Université du Québec à Montréal Centre de Neurosciences de la Cognition and Département de Psychologie Montréal Québec Canada; ^2^ Children’s Hospital of Eastern Ontario Psychology and Oncology Ottawa Ontario Canada; ^3^ Hôpital de Montréal pour Enfants Département de Psychologie Montréal Québec Canada; ^4^ Hôpital Notre-Dame Service d'Épilepsie Montréal Québec Canada; ^5^ Grand River Hospital Kitchener Ontario Canada; ^6^ Hôpital Marie-Enfant Neuropsychologie Montréal Québec Canada; ^7^ Hôpital Sainte-Justine Psychologie Montréal Québec Canada; ^8^ Hôpital Côte des Neiges Centre de Recherche Théophile Alajouanine Montréal Québec

**Keywords:** aetiology, lesion, brain, sex, sex difference, intelligence, recovery

## Abstract

Neurologists and neuropsychologists are aware that aging men are more at risk than women for brain damage, principally because of the well known male-predominant risk for cardiovascular disease and related cerebrovascular accidents. However, a disproportion in prevalence of brain damage between the sexes in childhood may be less suspected. Furthermore, sex-specific risk for other aetiologies of brain damage may be little known, whether in the pediatric or adult populations. Proposals of a sex difference in cognitive recovery from brain damage have also been controversial. Six hundred and thirty five “consecutive” cases with cortical focal lesions including cases of all ages and both sexes were reviewed. Aetiology of the lesion was determined for each case as was postlesion IQ. Risk was highly male prevalent in all age groups, with a predominance of cardiovascular aetiology explaining much of the adult male prevalence. However, several other aetiological categories were significantly male prevalent in juveniles (mitotic, traumatic, dysplasic) and adults (mitotic, traumatic). There was no sex difference in outcome (i.e., postlesion IQ) of these cortical brain lesions for the cohort as a whole, after statistical removal of the influence of lesion extent, aetiology and presence of epilepsy. Mechanisms potentially responsible for sex differences in prevalence, aetiology of brain damage, and recovery, are reviewed and discussed.

## 1. Introduction

The purpose of this investigation was to analyze sex differences in a large non-epidemiological cohort so as to determine whether there exists a sex difference in intellectual recovery from focal cortical brain lesions. Because numerous variables, other than the simple presence of the lesion itself, are potential sources of sex differences in intellectual outcome (age of onset, aetiology, lesion extent, etc.), it was felt that a very large and well characterized sample would allow us to statistically control extraneous variables. However, because there are several known sex differences in neuropathology such as risk factors for certain aetiologies,

ISSN 0953-4180/01/02/$8.00 © 2001/2002 – IOS Press. All rights reserved we deal with these issues in the first part of the literature review. The second part of the literature review deals with what is currently known about sex differences in recovery from brain damage.

### 1.1. Cardiovascular aetiology

Men are at greater risk than women for brain damage secondary to cardiovascular disease [12]. Smoking [43], alcohol consumption [43], hypertension [78], and type-A personality [14,38] are known male predominant risk factors. Androgens also exert pressure on the vascular system [53] while estrogen may have a mildly protective action [55]. We have not been able to determine whether these steroid actions are operant in the foetal or infant brain however. In the specific case of juvenile brain vascular anomalies, the evidence is to the effect of a higher male risk for brain vascular developmental anomalies [62] and intracranial hemorrhage [16], but not hemangioma [1] or Sturge-Weber syndrome [7].

### 1.2. Infectious aetiology

Infection of the brain can result, of course, in an identifiable and permanent brain lesion (cf., a tissue destroying abcess). Though the immune infrastructure of the human male is known to be less potent in several respects than the female's [69], it is not clear whether he suffers more frequent infections at equal exposure than the female in all circumstances, but there is some evidence to that effect [57]. Any immune disadvantage involving the blood-brain barrier or the immune infrastructure of the brain (cf., microglia: see [58] for evidence of a sex difference) would place that individual more at risk for brain damage of infectious aetiology. During gestation, risk for brain damage of vascular, mitotic and infectious aetiology could be mediated by immunity. This could explain some of the sex prevalence differences observed in cases assumed congenital but diagnosed at any age (it is not unusual for a porencephalic lesion, for example, to be identified serendipitously via a brain scan carried out for other reasons).

### 1.3. Mitotic aetiology

Most mitotic conditions of known genetic origin are autosomal. This would lead us to expect only minor sex differences in prevalence. However, a modulating role of steroid hormones (via immunity for example) cannot be excluded a priori. In fact it is known that some mitotic conditions of the brain are male prevalent and that others are female prevalent while others present an equal risk (cf., craniopharyngioma [13]). Brain cancer, all categories included, is known to be more prevalent in adult men than women [60] and in male than female children [64].

### 1.4. Traumatic aetiology

Young men are far more at risk for head trauma than any other age or sex category [22]. The sex difference is primarily due to automobile accidents and assaults. Young men drive more recklessly [67], are reckless pedestrians [63] and also physically assault and are physically assaulted [22] far more often than women. It is less well known that male prevalence of head trauma begins as soon as the phenomenon can be measured, that is, right after birth [6]. This discrepancy could consist of greater turbulence, explorativeness and fearlessness of the male infant and child [17] leading to falls and other types of accidents.

### 1.5. Unknown aetiology

For every 100 human females conceived, there are between 107 and 124 male conceptions. But the male embryo is more likely to spontaneously abort. As a result, for every 100 female births there are 106 male births. Factors leading to embryonic death could conceivably also lead to brain damage in those who survive. In light of this, it would not appear surprising that whether there is a genetic factor involved or not, prevalence of congenital porencephaly [79] is higher in the male sex.

### 1.6. Epilepsy

The physiopathology underlying epilepsy is often identified vaguely or not at all. It has been reported on several occasions that males are more at risk for epilepsy whether the onset be in childhood [52,66] or adulthood [20,70]. It would seem quite likely that brain lesions associated with epilepsy ought therefore to be more prevalent in the male sex, but this remains to be determined. Epilepsy commonly complicates recovery from focal brain lesions regardless of the aetiology of the brain lesion. For example, certain forms of epilepsy are associated with lower IQ. More specifically, "occurrence of regular seizures during a critical period in early childhood neural maturation poses the greatest risk to cognitive development in the epilepsy population" [23]. Thus, any investigation of sex or age differences in recovery from brain lesions should take epilepsy into account.

The preceding considerations underscore the complexity of judging, from clinical samples, whether there are sex differences in intellectual outcome from cortical lesions.

### *1.7. Sex differences in cognitive outcome of brain lesions*

It is well known that the human female has a better neurodevelopmental, cognitive, and school outcome following extreme premature birth [32,49–51] or extremely low birthweight [41]. The female sex also presents a clearly higher survival rate (48% versus 28%) after extremely low birth weight according to Hoffman and Bennett [31]. The direction of sex differences is similar in lesion experiments with infant primates with regard to frontal lesions [24]. Several studies have reported higher survival rates of female rats after pallidal or hypothalamic lesions (see [42]). The work of Goldman and colleagues is particularly relevant to the human literature in that they also found that cognitive recovery was significantly superior in female survivors. Groswasser and colleagues [27] found that a large cohort of female traumatic brain injury patients had much better cognitive recovery than males.

Rapin [61] reviewed effects and outcomes (including IQ) of unilateral lesions in a set of studies of children, and found that the female sex had a better prognosis. Other studies of post-lesion IQ in children had smaller groups and did not investigate sex differences [3,5, 72]. It is still not clear whether the sex difference in lesion outcome varies as a function of age of onset (i.e., whether there is anything developmentally specific about the effect), or whether the effect would be manifest despite controlling for lesion extent or lesion aetiology. In fact, sex differences with respect to these variables are known to exist and have been evoked as major sources of contamination of investigations purporting to compare the sexes with respect to recovery from brain lesions (see [9,10]).

McGlone [46] found that a smaller proportion of women (13%) than men (48%), presented with aphasia following a left hemisphere brain lesion. The men also had more verbal memory deficits than the women. Kimura [39] conducted an "ex post facto" investigation of aphasia and apraxia in 216 right handed patients with left hemisphere lesions. A higher proportion of men presented with aphasia than women. However no sex difference was observed in aphasia following stroke in a study by Hier and colleagues [30]. It is debated as to whether this female advantage is due to a different brain organization or to greater robustness of the female brain's ability to heal itself, or to an artefact imputable to methodological difficulties. A meta-analysis of previously published results has found that women have a better recovery from brain lesions than men on both the Performance IQ (PIQ) and on the Verbal IQ (VIQ) scales [35].

The studies reviewed in the two previous sections have methodological limitations. For example, in some studies, aetiologies of the lesions affecting each sex were obviously not comparable. The ages of onset and the intervals from lesion onset to IQ testing were not always sex-matched, lesion extent was most often completely ignored, and sex-biased complications known to affect IQ (e.g., epilepsy, see [74]) were not taken into account. Lesion location was not radiologically confirmed in all the studies. The studies might not have paid enough attention to possible modulation of cognitive recovery by lesion side. These issues are not trivial for several reasons: 1) the female sex is known to manifest certain superiorities in the verbal domain [36] while the male sex is known to present an advantage in the spatial domain [15], 2) there are known asymmetries of hemispheric physiology and of hemispheric anatomy which are sex-specific (see [11] for a review). Most investigations did not comprise enough subjects to control for any of these variables. Considering that in the Hier et al study, where the problem of gender bias in aetiology was circumvented by including only cerebrovascular cases, no sex difference in intellectuel recovery accrued, it seems that the issue of sex differences in recovery from brain damage deserves further scientific attention.

Mechanisms that protect a human female from deleterious consequences, or that favor a good prognosis, after a brain lesion, could be sociocultural or physiological. Sociocultural factors could include greater effort expended by the patient herself or her family in the search for compensatory resources (educational, remedial, emotional, etc.). We are unaware of any evidence in support of this possibility. The most likely candidates for sex-specific physiological protection factors would include either steroid-brain or immune-brain processes or interactions between these. Rodent experiments have shown that circulating steroid status of mammals definitely influences behavioral recovery. More specifically, estrogen appears to be a potent factor in recovery from cytotoxic brain lesions [21] or brain lesions induced by ischemia [18]. Evidence is accruing to the effect that one mediator of this estrogen neuroprotective factor involves the immune system [2]. Progesterone also seems to have a wide ranging neuroprotective function [4,25]. It protects against post-ischemic damage, and manifests numerous neuroprotective properties in vitro. Unfortunately, we were not able to find any evidence to the effect that estrogen or progesterone specifically protects a human female from the cognitive sequellae of brain lesions.

The purpose of this investigation was to better understand the modulating influence of gender in brain lesions and recovery therefrom, and thus to help refine the work of practicing neurologists and neuropsychologists.

## 2. Method

### 2.1. Subjects

Two hundred and ninety nine cases were drawn from the published literature (see Montour-Proulx, 2000 for details) and 336 from eastern Canadian hospital records (see Montour-Proulx, 2000 for details) answering to a set of inclusion and exclusion criteria. No effort was expended to recruit one sex more than the other.

Inclusion criteria were: 1) presence of a radiologically or surgically confirmed and explicitly localized unilateral cortical lesion involving at least one brain lobe (only the frontal, temporal, parietal and occipital lobes were considered), and subcortical damage was not an exclusion criterion provided cortical damage was clearly established. Considering that human cortex is only about 5 mm thick, pure cortical lesions must be very rare, 2) availability of FSIQ, PIQ and VIQ obtained from one of the Wechsler scales (WPPSI, WISC or WAIS), age at lesion onset, sex, aetiology of the lesion, lesion location, age at time of IQ testing. Age of onset was set at zero in cases judged "congenital" by the treating physician or the authors of the article. We reviewed all the cases ourselves, to determine possible congenital aetiology, using the reviews of congenital aetiologies of brain damage of Kleihus and Cavenee [40] and Spaycar and Randolph [68]: cavernous hemangioma, hypoplasia, dysplasia, macrogyria, pachygyria, polymicrogyria, microdysgenesia, porencephaly, arachnoid cyst, arteriovenous malformation, Sturge-Weber syndrome, dysembryoplastic neuroepithelial tumor, etc.

Table 1
Description of the lesions in terms of extent and location for the juvenile and adult groups

| | Juveniles | Adults |
|---|---|---|
| *Lesion extent* | | |
| 1 lobe involved only | 62% | 55% |
| 2 lobes involved only | 18% | 35% |
| 3 lobes involved only | 6% | 7% |
| 4 lobes involved | 14% | 3% |
| *Lesion location* | | |
| Frontal lobe involvement | 45% | 40% |
| Temporal lobe involvement | 68% | 49% |
| Parietal lobe involvement | 38% | 46% |
| Occipital lobe involvement | 22% | 22% |

Exclusion criteria were: 1) systemic disease (ex: leukemia, liver disease, cardiac disease, etc.), 2) CNS neuropathology other than the focal lesion (ex: trisomy, monosomy, etc.), 3) radiotherapy or chemotherapy. We rejected all cases that had any missing data whatsoever. We arbitrarily divided the cohort into "pediatric" (lesion onset $<$ 18 years of age, $N = 417$) and "adult" (lesion onset 18 years of age or more, $N = 218$) subgroups. The juvenile group had a mean age at symptom onset of $5.2$ years ($SD = 5.5$) and a mean age at the time of testing of $17.2$ years ($SD = 10$). The adult group had a mean age at symptom onset of $46.6$ years ($SD = 18$) and a mean age at the time of testing of $48.9$ years ($SD = 16.7$). See Tables 1 to 6 for further clinical description of these two groups. Note that the sample studied cannot be considered "epidemiological" in any way.

### 2.2. Procedure

For the purpose of investigating sex-specific prevalences of aetiologies, we created a six category classification of the aetiologies (see Table 2 for a breakdown by age-group and sex). For the sake of convenience, we present incidence of epilepsy in Table 2 as well. Each case was given at least one and at most three aetiological categories (or epilepsy) in this system. Thus, a minimum of 635 aetiological tags and a maximum of 1905 could have been derived: in fact, there were 1065 tags altogether. Tallies of these aetiologies (and epilepsy) per sex and age-group were submitted to Chi square analysis. Age at the time of lesion onset was fixed on the basis of the aetiology occuring at the youngest age.

For the purpose of comparing the sexes with regard to recovery (postlesion IQ), we first determined whether the sexes differed with regard to lesion extent (defined as number of lobes lesioned from 1 to 4), age-of-onset, age-at-testing, lesion site, lesion-onset-to-IQ testing in-

Table 2
Frequencies of aetiologies of the brain lesions, and of epilepsy, as a function of age-group and sex

| Aetiology/ Age group | Infectious | Vascular | Mitotic | Dysplasic | Traumatic | Other | Epilepsy |
|---|---|---|---|---|---|---|---|
| *Juvenile onset* (< *18 years*) | | | | | | | |
| Male | 12 | 29 | 58 | 37 | 65 | 22 | 136 |
| Female | 17 | 24 | 27 | 23 | 34 | 14 | 109 |
| Chi2/P | 0.9/NS | 0.5/NS | 11.3/0.001 | 3.3/NS* | 9.7/0.002 | 1.8/NS | 3.0/NS* |
| *Adult onset* (> *18 years*) | | | | | | | |
| Male | 5 | 86 | 23 | 2 | 40 | 158 | 24 |
| Female | 2 | 32 | 10 | 0 | 7 | 60 | 9 |
| Chi2/P | –/– | 24.7/0.001 | 5.2/0.02 | –/– | 23.2/0.001 | 44.0/0.000 | 6.8/0.009 |

Note. Epilepsy is not an aetiology for a brain lesion, but rather a complication. The aetiological category "other" is primarily composed of cases with vague diagnoses such as "atrophy", "local sclerosis", "anoxia", "prematurity", "forceps injury", "encephalopathy", "gliosis", "cyanosis", "obstetric complications", etc., in decreasing order of frequency.
*Effect significant only at one tail. NS = Non-significant, one tailed test.

terval, clinical severity (static aetiology, static aetiology + epilepsy, progressive aetiology, progressive aetiology + epilepsy). See Tables 2 and 3. Having established that some of these variables differed as a function of sex and were indeed correlated with our measures of recovery (Full Scale IQ, Verbal IQ, Performance IQ), we proceeded to include these in a MANCOVA and ANCOVA models comparing the two age groups and the two sexes on the IQ measures. Of course, we supposed that the sexes and age groups were of comparable IQ at outset -an assumption justified by excellent age norms for these IQ tests and absence of sex differences on FSIQ, VIQ and PIQ scores [75–77].

## 3. Results

### 3.1. *Frequency breakdowns by sex, age-group and aetiology*

The male sex ($N = 404$) was far more at risk for a brain lesion than was the female sex ($N = 231$) (Chi$^2 = 47.1$, $p = 0.001$).

The distribution of frequencies of aetiologies is presented in Table 2 as a function of sex and age-group. Epilepsy, which in these cases has to be considered a "complication", is also tallied by sex in Table 2. Chi$^2$ tests served to compare the sexes separately for each age group and for each aetiological category.

### 3.2. *Sex differences in cognitive outcome of brain lesions*

We were surprised to discover that for the cohort as a whole the sexes were not comparable on several other variables potentially capable of modulating IQ scores (lesion extent: Chi$^2 = 17.2$, $p = 0.001$, lesion onset to IQ testing interval: $F(1, 633) = 16.5$, $p = 0.0001$, clinical severity: Chi$^2 = 13.2$, $p = 0.004$). These sex differences are presented in Table 3.

We then ascertained the extent to which a relation existed between these potentially confounding variables (i.e., lesion extent, lesion onset to IQ interval, clinical severity) and the IQ measures (FSIQ, VIQ, PIQ). Because the aetiology variable (static, static with epilepsy, mitotic, mitotic with epilepsy) held a very curvilinear relationship with IQ, we broke it down into four dichotomous variables. To our surprise, epilepsy negatively affected IQ more than a progressive (mitotic) condition. Most of these potential confounds were significantly correlated with one or several IQ measures. See Table 4.

To ascertain whether the sexes differed with regard to lesion site, we first carried out Chi Square tests on four lesion site variables as a function of sex. Lesion site was operationalized as follows: if the frontal lobe was involved in the lesion this generated a tally for that lobe, and so on for the other lobes. Thus the lesion site variable was a four level variable with total frequencies amounting to far more than 635. We completed a Chi Square analysis of this lesion site variable as a function of sex. This analysis revealed no significant interaction between sex and the frequency profile of lobes involved in the lesion. However, as the individual lesion sites (frontal, temporal, parietal and occipital) were significantly correlated with the three IQ measures, we opted to include the lesion site variable in the analyses.

Analyses of variance were carried out using SAS v6, GLM procedure. So as to minimize type-2 error, we first subjected the data base to the fullest possible statistical model, a $2 \times 4 \times 2 \times 2 \times 2$ MANCOVA (sex, lesion site, hemisphere-lesioned, age-group, IQ-type),

Table 3
Age at lesion onset, age at the time of intelligence testing, lesion extent, severity of the aetiology, as a function of age-group, hemisphere-lesioned and sex (means and SDs)

| | Age of lesion onset (years) | Age at time of IQ testing (years) | Lesion extent* | Severity of the aetiology* |
|---|---|---|---|---|
| Juvenile Left hemisphere Female ($N$ = 95) | 4 (5.3) | 18 (10.7) | 1.9 (1.2) | 1.9 (0.9) |
| Juvenile Left hemisphere Male ($N = 135$) | 5 (5.7) | 17 (10.3) | 1.7 (1.0) | 2.0 (1.1) |
| Juvenile Right hemisphere Female (76) | 6 (5.3) | 19 (10.0) | 1.8 (1.2) | 2.0 (0.9) |
| Juvenile Right hemisphere Male ($N = 111$) | 6 (5.6) | 16 (8.6) | 1.5 (0.9) | 2.1 (1.0) |
| Adult Left hemisphere Female ($N = 27$) | 49 (17.5) | 50 (16.6) | 1.4 (0.7) | 1.4 (0.9) |
| Adult Left hemisphere Male ($N = 71$) | 43 (16.8) | 46 (16.3) | 1.6 (0.7) | 1.5 (0.9) |
| Adult Right hemisphere Female ($N$ = 33) | 44 (18.6) | 48 (16.7) | 1.6 (0.8) | 1.5 (0.8) |
| Adult Right hemisphere Male ($N = 87$) | 50 (17.9) | 51 (16.7) | 1.6 (.8) | 1.4 (0.8) |

*See the Method section for the operationalization of these variables.

with as dependent measures Verbal IQ (VIQ) and Performance IQ (PIQ). In the MANCOVA and ANOVAs which follow, a lesion site effect was always tested with the frontal*parietal*temporal*occipital term. The IQ-type factor was the only repeated factor. The covariables, the variance of which we partialed out, were lesion extent, progressive aetiology, and epilepsy. This is accomplished in GLM using the appropriate sum of squares term. Variables with no effect on IQ measures (onset to test interval, static aetiology, progressive aetiology + epilepsy) were excluded from the model. The category static aetiology + epilepsy was excluded because of partial redundency with the epilepsy covariate. The effects of interest for this report concern only the sex variable. This represents a very complex design: though there were no missing data, replicates were lost in the GLM production of matrices, such that the MANCOVA and ANCOVAs reported next are based on 544 subjects. In the MANCOVA there was no main effect of sex. Significant interactions involving sex were IQ type $x$ age group $x$ sex $((F2, 544) = 16.3,\ p < 0.0001)$, IQ type $x$ hemisphere lesioned $x$ sex $((F(2, 544) = 13.3,\ p < 0.0001)$, IQ type $x$ lesion site $x$ sex $((F(22, 544) = 2.2,\ p < 0.003)$, IQ type $x$ lesion site $x$ hemisphere lesioned $x$ sex $((F(22, 544) = 2.4,\ p < 0.0003)$, IQ type $x$ age group $x$ hemisphere lesioned $x$ lesion site $x$ sex $((F(30, 544) = 2.0,\ p < 0.0003)$. See Tables 5 and 6 for the means. An identically formated ANCOVA on Full Scale IQ revealed no effect of sex at all. The same analyses without correction yielded all the same interactions, with the addition of a lesion site $x$ sex effect on Full Scale IQ $((F(23, 544) = 4.5,\ p < 0.0001)$. ANCOVA with the same correction for covariates as previously on Verbal IQ yielded two significant effects involving sex, a hemisphere lesioned $x$ sex interaction $((F(2, 544) = 4.0,\ p < 0.02)$ and a lesion site $x$ sex interaction $((F(2, 544) = 2.0,\ p < 0.01)$. See Tables 5 and 6 for details. The same analysis of Performance IQ also yielded two significant effects involving sex, an age group $x$ sex interaction $((F(2, 544) = 6.4,\ p < 0.002)$ and a hemisphere lesioned $x$ lesion site $x$ sex interaction $((F(22, 544) = 2.1,\ p < 0.003)$. See Tables 5 and 6 for details.

We ascertained that by removing lesion site from the MANCOVA and ANCOVA models, we lost none of the 635 subjects. We completed all the same analyses as above without the lesion site independent variable.

Table 4
Correlations between various "clinical" variables and Verbal IQ, Performance IQ and Full Scale IQ ($N = 635$)

| | Full Scale IQ | Verbal IQ | Performance IQ |
|---|---|---|---|
| Age of onset | 0.08* | 0.19*** | −0.09* |
| Age at time of testing | 0.08 | 0.21*** | 0.11** |
| Onset to test interval | −0.03 | −0.04 | −0.01 |
| Static aetiology | 0.02 | 0.06 | −0.06 |
| Static aetiology + epilepsy | −0.06 | −0.09** | 0.02 |
| Progressive aetiology | 0.12** | 0.09* | 0.12** |
| Progressive aetiology + epilepsy | −0.05 | −0.05 | −0.05 |
| Epilepsy | −0.02 | −0.05* | −0.04 |
| Lesion extent | −0.32*** | −0.25*** | −0.33*** |

Note. $^{*}p < 0.05$, $^{**}p < 0.01$, $^{***}p < 0.001$. Relations with dichotomous variables are calculated using the point biserial coefficient and with continuous variables using the Pearson product-moment coefficient.

Table 5
Mean intelligence quotients (FSIQ, VIQ, PIQ) as a function of age-group (± 18), hemisphere lesioned and sex $N = 544$)

| | Juveniles | | Adults | |
|---|---|---|---|---|
| | Left hemisphere lesions | Right hemisphere lesions | Left hemisphere lesions | Right hemisphere lesions |
| *Full Scale IQ* | | | | |
| Females | 91 | 87 | 94 | 95 |
| Males | 92 | 94 | 92 | 92 |
| *Verbal IQ* | | | | |
| Females | 91 | 89 | 95 | 101 |
| Males | 91 | 94 | 94 | 99 |
| *Performance IQ* Females | 92 | 87 | 94 | 89 |
| Males | 94 | 95 | 92 | 83 |

There was no sex main effect nor any two way or three way interactions involving sex. Because the brain is commonly believed to manifest loss of plasticity by age five [45], we carried out the same MANCOVAs and ANOVAs as above with age groups defined as younger and older than five years. There was again no sex main effect nor any two or three way interactions involving sex.

## 4. Discussion

### 4.1. *Sex, age at the time of lesion onset and aetiologies of focal cortical lesions*

There was a spectacular male prevalence of cases of brain lesion in both the juvenile and adult onset subgroups. This sex difference was due to a greater male prevalence of mitotic and traumatic aetiologies in both age groups as well as a greater male prevalence of vascular aetiologies in the adult group. Of course, this conclusion must be considered in light of the fact that mortality from such lesions could also vary as a function of sex and thus influence the above mentioned prevalences -especially in the most elderly range. We had no access to the mortality in the present study. An interesting study by Gordon and Rosenthal [26] found that the male sex was indeed at greater risk for mortality after a stroke (× 1.6) in a hospital setting. This would predict then that our distributions are biased by an over-representation of women -considering that collecting IQ after a lesion requires the subject to have survived. In short, the male prevalences in all of the diagnostic categories of the present study could be underestimates, and the male prevalence in the vascular category is most probably an underestimate.

As expected, the male sex was more at risk for epilepsy as a complication (one tailed only in the juvenile onset subgroup, and two-tailed in the adult onset subgroup). Infectious and dysplasic aetiologies were too rare to support inference testing in the adult onset subgroup. An epidemiological investigation of sex distributions of brain lesions of infectious origin would be of great help.

It is notable (and perhaps as of yet unheard of) that brain anomalies (lesion-like) of dysplasic aetiology were indeed male prevalent (though only one tailed) in the juvenile group. This may relate, for example, to the debate over whether dyslexia is truly male prevalent (or simply over-diagnosed in the male sex) and whether the male prevalence is due to the now frequently postmortem confirmed dysplasic elements (brain warts or ectopias, vascular dysplasia, heterotopias, all indicative of neural migrational aberrations). Postmortem analysis of the brains of three female dyslexics revealed presence of exactly the same dysplasias as reported in all previous male cases [34]. We suggest that congenital dyslexia may simply be a special case of more widespread male-prevalent risk for neural dysplasia. Epidemiological research on brain lesions of dysplasic aetiology would be helpful in clarifying this specific issue in a wider context.

The absence of a sex difference in prevalence of vascular cases in the juvenile onset subgroup and the highly significant male prevalence in the adult onset subgroup would be compatible with neuroprotective female steroid factors involving progesterone and/or estrogen or a male steroid (testosterone) vascular stress factor and/or a lifestyle factor (higher incidence of smoking, drinking, stress or type-A personality in men) -the first two of these factors being less operant, in principle, prior to puberty and the last obviously being less so in fact. Important research needing to be done on neuroprotection by steroids could include, we think, an animal study of brain lesion effects (survival, and also cognition) as a function of multiple hormone levels during pregnancy in females. Estrogen climbs to a very high level just before parturition.

The ensemble of the present findings is consistent with the mechanisms of gender-specific risk outlined in the introduction.

### 4.2. *Sex differences in intellectual outcome of brain lesions*

It cannot be claimed from these results that there exists a generalized sex difference in cognitive recovery from brain lesions. On the contrary, for the cohort as a whole, none of the IQ scores differed in a global manner as a function of sex after correction for relevant "confounding" variables (lesion extent, progressive aetiology, lesion site and epilepsy). Considering that the subjects' age, hemisphere lesioned, lesion site and the IQ subscale all interacted with the sex variable, it can easily be conceived how previous researchers could have come upon a biased effect of sex due to inadequate control of any of the above variables.

The significant interactions obtained here, involving sex, do suggest however that male and female brains may organized differently with regard to certain cognitive functions. Recall that all the MANCOVA interactions inolved IQ type. In other words, male and female brains do not seem to have the same cognitive profile after a lesion. The more interpretable effects are the two way interactions observed in ANCOVA on one or the other subscale of the Wechsler intelligence tests. With regard to Verbal IQ the hemisphere lesioned x sex interaction indicates that females recover better from left hemisphere lesions than right hemsphere lesions while males do the reverse (see Table 5). Again with regard to Verbal IQ, the lesion site x sex interaction is harder to interpret because the lesion site variable is unpure and because it generates small cells (Table 6). We prefer to abstain from interpretation of this effect. With regard to Performance IQ the age group $x$ sex interaction reflects the fact that the women do better than the girls whereas the boys do better than the men (see Table 5). Again with regard to Performance IQ, the hemisphere lesioned $x$ lesion site $x$ sex effect (see Tables 5 and 6) will do without our interpretation for the same reasons as above.

In short, we suggest that this investigation challenges the perhaps dominant (but certainly controversial) belief that the human female can expect a better cognitive outcome after incurring a brain lesion. The credibility of this challenge, we think, is based on the large number of cases reviewed here and on the opportunity we had of controlling for several potentially "confounding" variables. Supposing then that there is no sex difference in intellectual recovery from brain lesions, what is to be made of other sex differences in recovery from disease? Why does the human female present better cognitive outcome of extreme prematurity and better survival from stroke in old age? Why do female monkeys present better cognitive recovery from focal lesions and not human females? These are mysteries that call for further research.

The results of the present investigation are compatible, in a sense, with most of the relevant scientific literature. The female with adult lesion onset seems to have the better cognitive outcome [35,39,46]. However, this effect is negated when childhood onset cases are included in the sample (see Table 5). A dominant theme in the literature from the 70s on sex differences in cognitive recovery from brain lesions was that women have a better recovery of language (see the introduction). We have since ceased to believe in this sex difference in recovery of language functioning in the 80s based on more reliable empirical evidence [37,65,73].

It should perhaps not be surprising that if one sex is going to benefit from better healing of the brain after a lesion, the effect should be most manifest on PIQ and

Table 6
Mean intelligence quotients (FSIQ, VIQ, PIQ) and number of cases as a function of lesion site and sex

| | Frontal lobe lesions ($N = 103$, 171) | Parietal lobe lesions ($N = 95$, 162) | Temporal lobe lesions ($N = 159$, 234) | Occipital lobe lesions ($N = 59$, 82) |
|---|---|---|---|---|
| *Full Scale IQ* | | | | |
| Females | 85 | 95 | 90 | 85 |
| Males | 90 | 95 | 91 | 87 |
| *Verbal IQ* | | | | |
| Females | 88 | 88 | 92 | 90 |
| Males | 93 | 91 | 93 | 92 |
| *Performance IQ* | | | | |
| Females | 84 | 82 | 90 | 85 |
| Males | 89 | 87 | 91 | 87 |

Note. The lesion is not necessarily "limited" to a single lobe. Full Scale IQ is not an average of the two subscales, it is derived from its own norms.

less so on VIQ. Indeed, PIQ is simply more sensitive to brain damage in general [8,44,59] and should thus be more sensitive to other variables that impede or facilitate brain healing as well (lesion extent, aetiology, epilepsy, etc.). In the present investigation, VIQ was highly significantly superior to PIQ for the cohort as a whole $(F(1,632) = 19,\ p = 0.0001)$, though all the variance came from the adult cohort. The reasons are well known: Performance IQ measures more "fluid" intelligence (i.e., novel manipulation of unusual material) under time pressure, whereas Verbal IQ measures more "crystallized" intelligence (i.e., routine manipulation of overlearned material) without the pressure of time. Whereas the difference in content (i.e., verbal versus visuospatial) between the Verbal and Performance IQ scales is presumed equally brain-dependent, these other differences are not [30]. In children, verbal intelligence is probably not yet cristallized enough to show better recovery after brain lesions than non verbal intelligence. In the present investigation, prior to statistical correction for the covariates, it was PIQ which best discriminated the sexes, giving a small and very localized advantage to females.

Investigation of sex differences in cognitive recovery from juvenile onset lesions has so far been very limited. The present results challenge the findings of Rapin [61] who found a better outcome in girls than in boys. The present results argue that general intellectual ability (and recovery) seem to be identical for the two sexes regardless of their age. Incidentally, it has been argued that plasticity of intellectual recovery is limited to the first few years [45]. This point of view has since been challenged: for example, age at the time of hemispherectomy does not have much bearing on intellectual recovery [72]. The results of the present study indicate that whether this developmental effect exists or not, it is apparently not related to gender.

In short, the sexes differ radically in risk for various serious conditions of brain development and ageing, leading to brain lesions: males are more at risk. In the present sample the sexes differed significantly from each other with regard to aetiology and extent of the lesion, and with regard to risk for epilepsy. On the other hand, among survivors of such lesions, neither sex appeared to benefit remarkably or consistently from better intellectual recovery after a brain lesion (other aspects being equal). The implication of these findings for clinical practice is that both sexes are equally meritorious for cognitive-perceptual rehabilitation after focal brain lesions, despite the fact that the male sex may require some more medical attention because of the greater number of patients. Selection of more sensitive neuropsychological measures, of a wider range of recovery periods (for example measured in days after the lesion) and more refined estimation of lesion sites, as would be possible only in a large prospective multicenter study, could provide important vistas that are missing from the current report.

## Acknowledgements

This research was made possible partly by a Natural Sciences and Engineering Research Council grant of the governement of Canada to the first author, a Fonds Concerté d'Action et de Recherche grant and a Fonds de Recherche en Santé du Québec grant from the government of Quebec to the first author.
